# Development of a Computer Program for Determining the Dose of Laser Radiation (860 nm) Received by Tumor and Breast Tissue

**DOI:** 10.3390/cancers18030442

**Published:** 2026-01-29

**Authors:** Vladimir Alexander Mikhaylov, Nadezhda Voltchenko, Dmitry Mikhailov, Vladimir Gladyshev, Evgene Sharandin

**Affiliations:** 1 Eternity Medicine Institute, Ground Floor, KG Tower, Marsa, Dubai Marina, Dubai, United Arab Emirates; 2 P.A. Herzen Moscow Scientific Research Oncological Institute, 125284 Moscow, Russia; 3 N.V. Sklifosovsky Institute of Clinical Medicine, Sechenov University, 119991 Moscow, Russia; 4 Faculty of Fundamental Sciences, Bauman Moscow State Technical University, 105005 Moscow, Russiashar@bmstu.ru (E.S.)

**Keywords:** low-level laser therapy (LLLT) (860 nm), mammary gland, absorption and scattering tissue, computer program

## Abstract

Laser therapy (860–910 nm) has been used to treat cancer since 1988. A key challenge is determining the dose of laser radiation absorbed by the tumor. Attenuation coefficients of laser radiation were determined for different breast tissues at various tissue thicknesses and beam angles. These results enable the development of a methodology for determining optimal irradiation modes by considering both the absorbed dose of laser radiation and objective examination data (ultrasound, mammography, CT, and MRI).

## Simple Summary

LLLT is a non-invasive treatment method for various pathologies based on the exposure of target tissues to an 860–910 nm laser beam. This approach has proven effective both as a standalone treatment and when combined with other therapeutic modalities. However, determining the exact dose of laser radiation absorbed by target tissue has remained challenging. Leading medical and technical organizations in the Russian Federation conducted experimental measurements of angular intensity distribution to determine attenuation and scattering coefficients for breast tissue. This substantially improved the accuracy of assessing laser radiation doses absorbed by both pathological breast tissue and surrounding tissues. Based on this research, a method was developed for determining the absorbed dose of laser radiation by the tumor based on objective examination of breast tissue (ultrasound, mammography, CT, and MRI). A computer program was developed to calculate the laser dose delivered to target tissues using an optical model of a multilayer scattering medium.

## 1. Introduction

Breast cancer ranks among the most prevalent oncological diseases worldwide. Currently, non-invasive methods, such as low-level laser therapy (LLLT), are being utilized in the treatment of this disease and its associated complications [1]. Numerous studies have investigated how LLLT with various wavelengths (482–860 nm) influences breast cancer and the complications identified following its treatment. Experimental research has confirmed the feasibility of applying this therapeutic modality in breast cancer care.

With an expanded understanding of the impact of laser radiation on tumor cells, it has become possible to employ LLLT both as a standalone treatment method [2,3,4,5,6] and in combination with other therapies [7,8,9,10]. Due to the prevalence of treatment-related complications, research has been conducted on the effects of LLLT on breast-cancer-related lymphedema [11,12,13,14,15,16,17,18,19], mucositis, and dermatitis [9].

Treatment concepts and methods have been modified over more than 30 years for various localizations, including breast cancer [20]. However, the problem of determining the radiation dose that tumors receive remained unsolved. Consequently, laser therapy has been applied in the absence of precise quantitative data, relying on empirical dose adjustment for individual cases. Under such conditions, treatment was performed without accurate determination of the laser dose absorbed by tissues along the beam propagation path.

Numerous publications document the scattering and absorption coefficients of laser radiation across a wide spectral range for various tissues, including human blood, bone (skull, ribs), skin, and epidermis. However, data for other tissue types remain limited [21]. Most importantly, measurements of the angular distribution of radiation scattered by tissues are nearly absent. Existing scattering diagrams are primarily derived from Monte Carlo simulations based on Rayleigh and Mie scattering theories. This lack of experimental data prompted the measurement of attenuation coefficients of laser radiation by breast tissues and the angular distribution of radiation scattered by tissues (scattering patterns).

To refine the methodology, an experimental study was conducted to investigate the scattering and absorption of laser radiation by porcine tissues. Based on these results, a computer program was created to calculate irradiation doses to pathological lesions. Based on this concept, the present study was initiated, drawing on principles and experimental findings obtained during investigation of the optical properties of porcine biological tissues. We advanced this approach by applying it to the clinically significant problem of laser dosimetry. The breast was selected as a model organ because it comprises several optically distinct tissue types and frequently contains pathological lesions amenable to laser irradiation. Additional considerations included the epidemiological significance of breast disease and encouraging clinical data regarding the efficacy of low-level laser therapy (LLLT) in treating various pathologies. These combined factors provided the foundation for developing a novel software tool that enables a new paradigm in planning and optimizing laser therapy. The implementation of this program has advanced clinical laser therapy to a new level of precision and personalization. The proposed approach expands current understanding of laser therapy in clinical practice and establishes a platform for ongoing development of individualized laser treatment protocols.

## 2. Rationale for Using the Research Method

Biological tissues are absorbing media with pronounced optical inhomogeneity. When radiation propagates in the tissue, it is repeatedly scattered by these inhomogeneities and partially absorbed. This results in broadening of the radiation beam and decreased intensity as it propagates. The lowest tissue absorption of laser radiation is observed in the near-infrared range (700–1400 nm). This is due to lower absorption by the main tissue components (water, hemoglobin, lipids). In this wavelength range, scattering prevails over absorption, and the penetration depth can reach 10 mm or more [22,23].

The angular distribution of the intensity of radiation scattered on optical inhomogeneities in tissues can be obtained within the framework of optical radiation transfer theory. Assuming that the medium has a random distribution of optical inhomogeneities without spatial correlation, describing radiation propagation requires three key parameters: the absorption coefficient *μ_a_*, scattering coefficients *μ_s_*, and the mean cosine of the scattering angle *g*. The values of these coefficients can be determined from measurements of collimated transmittance and scattering patterns of laser radiation in biological tissues [24] by solving the inverse problem (e.g., using the inverse Monte Carlo method) [25].

## 3. Materials and Methods

All experimental results were obtained in the Fundamental Sciences Faculty of the Bauman Moscow State Technical University (headed by Prof. Gladyshev V.O.). The laboratory staff has extensive experience in laser development [26] and high-precision optical measurements [27].

The studies were carried out on a specialized test bench (Prof. Gladyshev V.O., Sharandin E.A., Mikhailov D.V.), which enabled measurement of the attenuation coefficient and the angular dependence of radiation intensity scattered by tissue samples.

Pathological tissue samples obtained during surgery and collected for routine histological examination were also used in this study. The tissue samples were obtained within 3–5 h after surgery and transported in a special medical container at +4–8 °C to preserve biological integrity and tissue structure. All samples were standardized to dimensions of 1.5 × 1.0 cm with thicknesses ranging from 0.1 to 1.0 cm. To measure the scattering parameters, tissue samples of varying thicknesses were used.

**Figure 1:**
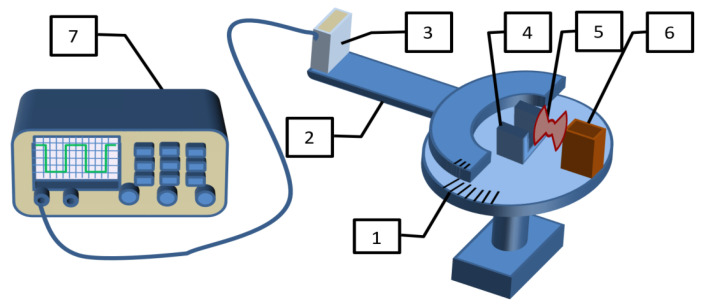
Scheme of the apparatus for measuring the scattering parameters of biological tissues. 1—turntable on a fixed base; 2—rotary platform with 180° rotation angle; 3—photodetector; 4—assembly for fixing tissue samples; 5—optical modulator; 6—semiconductor laser emitter with a wavelength of 860 nm; 7—oscilloscope for signal registration.

An experimental apparatus was designed for this purpose, as shown schematically in Figure 1.

A semiconductor laser emitter (6), an optical modulator (5) and an assembly for fixing tissue samples (4) were installed on the fixed base (1) of the turntable. A photodetector (3) was fixed on a rotary platform (2), with the detected signal being recorded by an oscilloscope (7). The laser emitter (860 nm) was equipped with a collimator that directed radiation perpendicular to the rotation axis of the rotary platform, where the sample was positioned. The incident beam diameter was approximately 0.4 mm in the horizontal plane and 3 mm in the vertical plane. The distance between the tested sample and the photodetector was 26 cm. The laser irradiance during measurements was typically maintained within the range of 10–30 mW and adjusted according to the tissue type being examined. The power level was carefully selected to keep the photodetector output voltage in the optimal range of 10 mV to 2 V across all angular positions during the measurement sequence. This configuration guaranteed linear operation of the photodetector’s current-voltage characteristic with a minimum signal-to-noise ratio of 4:1. When laser power was increased beyond 50 mW, significant thermally induced modifications of the tissue optical parameters were observed during the measurement procedure, necessitating the use of lower power levels. Between the sample and the laser emitter, a rotating modulator disk with two sectoral apertures was positioned. When the disk blocks the laser beam, the signal from the photodetector is proportional to the level of background radiation (the photodetector registers only the intensity of the surrounding light). When the laser beam passes through an aperture, the signal represents the combined intensity of scattered laser radiation and background illumination. Consequently, the oscilloscope displays a rectangular waveform with amplitude proportional to the intensity of scattered laser radiation. The total diffuse transmittance of tissue samples was determined by comparing the results of measurements of tissue scattering patterns with reference measurements for a calibrated diffuse scattering plate.

When a collimated laser beam is normally incident on biological tissue along the *x* axis, the radiation intensity decreases exponentially due to surface reflection, scattering, and absorption.

(1) $$I\left(x\right)=\left(1-R\right)I_{0}\exp\left(-\mu_{t}x\right)$$

where *I*_0_—the intensity of the radiation incident on the tissue, *R*—Fresnel reflection coefficient from the sample surface at normal incidence, *μ_t_*—extinction (attenuation) coefficient, equal to the sum of absorption coefficients *μ_a_* and scattering *μ_s_*, of the sample of thickness *x.*

If measurements are carried out for layers of different thicknesses from the same tissue sample, then, due to the equality of their reflection coefficients, it is possible to find the radiation attenuation coefficient by the following formula:

(2) $$\mu_{ti}=\frac{ln(\frac{I_{ij}}{I_{ik}})}{x_{ij}-x_{ik}}$$

where *i*—sample number, *j* and *k*—layer numbers of the studied sample, *I_ij_* and *I_ik_*—intensities of radiation beam along the axis through *j*-th and *k*-th layers of thicknesses *x_ij_* and *x_ik_*, respectively.

To determine the attenuation coefficient, each sample was cut into multiple layers of varying thicknesses. For each layer, the transmittance coefficient was calculated, and average values were determined.

The attenuation coefficient values *μ_t_* enable determination of the radiation intensity distribution propagating along the laser beam axis in biological tissues.

## 4. Results

### 4.1. Attenuation Coefficient Measurement Results

In the near-infrared range, biological tissues exhibit anisotropic scattering characterized by high directivity in the scattered radiation from thin samples. This behavior is likely due to the presence of large cell organelles (mitochondria, lysosomes, etc.). However, the diffuse approximation turns out to be inapplicable in the vicinity of the sample surface or in case of its small thickness. Consequently, the dependence of the attenuation coefficient on sample thickness was investigated in advance.

**Figure 2:**
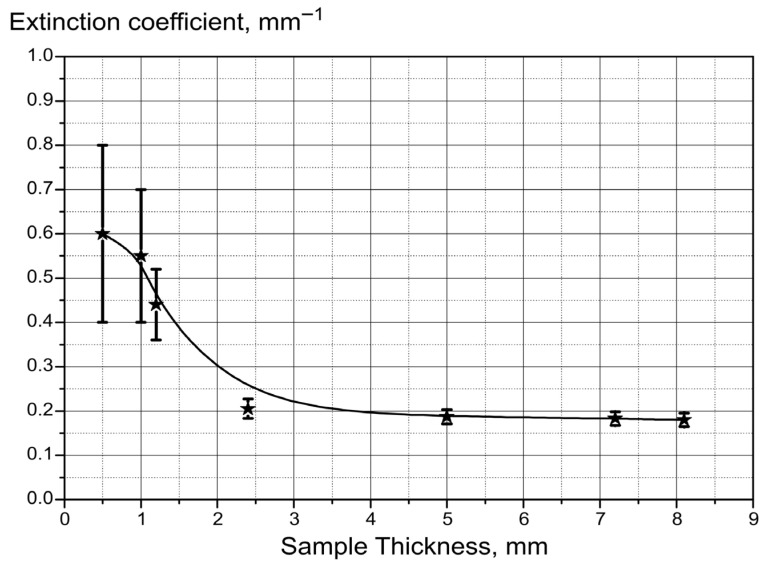
Dependence of attenuation coefficient on sample thickness. The asterisks on the graph indicate the experimental values. Vertical strokes indicate the range of measurement errors. The solid line is the result of the approximation of experimental values by a power polynomial using the least squares method.

Figure 2, as an example, illustrates the dependence of the attenuation coefficient on the thickness of the breast tissue sample. The graph shows a noticeable increase in the attenuation coefficient at small sample thicknesses. At these thicknesses, the measurement results were highly sensitive to sample positioning, leading to large errors in the recorded transmitted radiation intensity. To minimize these errors, only samples with thicknesses of at least 2 mm were used when determining the extinction coefficients.

Determination of the blood attenuation coefficient was used as a reference to assess the reliability of the obtained data. Analysis of the scattering measurement results showed a blood attenuation coefficient of 0.97 ± 0.06 mm^−1^, which agrees well with spectrophotometer data 0.99 mm^−1^. The obtained results are in good agreement with the data of other authors [28,29,30].

### 4.2. Breast Tissue Testing

Studies conducted by various authors have shown that for breast tissue the attenuation coefficient is in the range of 0.2–1.5 mm^−1^ at a wavelength of approximately 860 nm. A more precise value depends on tissue composition: adipose tissue exhibits lower attenuation coefficients (0.11–0.7 mm^−1^), while glandular and connective tissues show higher values (up to 1.5 mm^−1^) [31,32,33]. The differences in attenuation coefficients between glandular and connective tissues and breast glandular tissue likely reflect structural variations between these tissue types. The higher attenuation coefficient in breast tissue is attributable to its complex anatomical structure, which includes ductal systems and tubular glands. This structural complexity creates greater optical resistance to laser beam propagation through breast tissue compared to the more homogeneous and less dense glandular and connective tissues.

**Figure 3:**
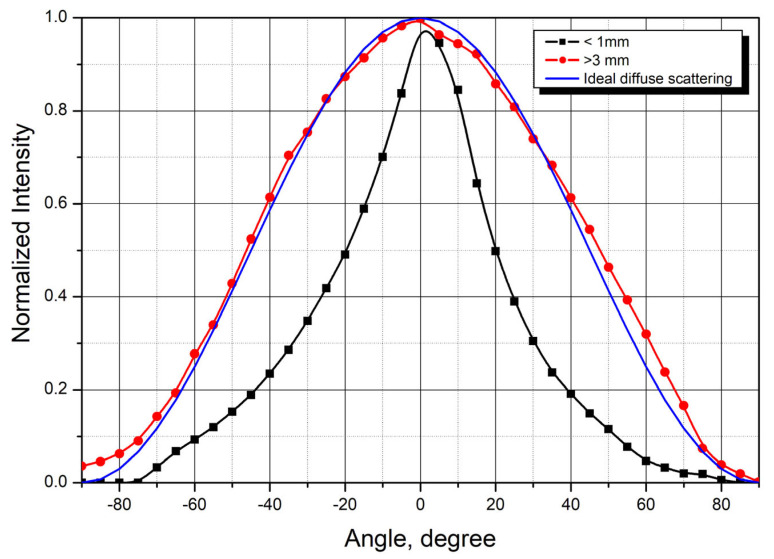
Scattering patterns from healthy breast tissue samples of different thicknesses (averaged over multiple samples) and reference diffuse scattering distribution.

To more accurately determine laser radiation absorption by tissues, angular scattering distributions were measured. Angular distributions of laser radiation scattered by healthy breast tissue samples are shown in Figure 3. The graphs were generated by averaging the measurement results from similar tissue samples (4 samples with thickness less than 1 mm and 15 samples thicker than 3 mm).

For thicknesses above 3 mm, the scattering pattern shows minimal change and approaches that of ideal diffuse scattering. Conversely, at small sample thicknesses, the scattered radiation maintains relatively high directivity.

**Figure 4:**
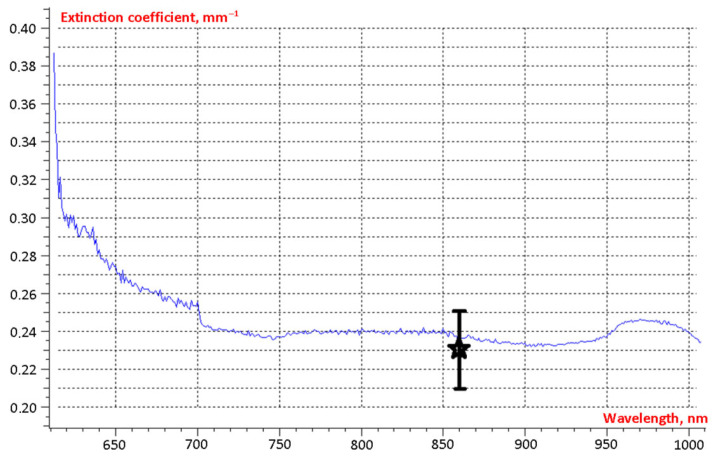
Results of attenuation coefficient measurements for breast tissue sample № 2024-11-19.

For № 2024-11-19 healthy tissue sample, the attenuation coefficient, similar to that for blood, was determined using two independent methods. Figure 4 shows the spectral dependence of the attenuation coefficient obtained using the spectrophotometer SF-56. The result obtained from the scattering measurement apparatus, including its error range, is marked with an asterisk. The measurements from both methods agree within the measurement uncertainty.

The attenuation coefficient for breast tissue samples typically ranged from 0.2 to 0.3 mm^−1^, with a mean value of 0.23 mm^−1^.

### 4.3. Breast Tumor Tissue Testing

**Figure 5:**
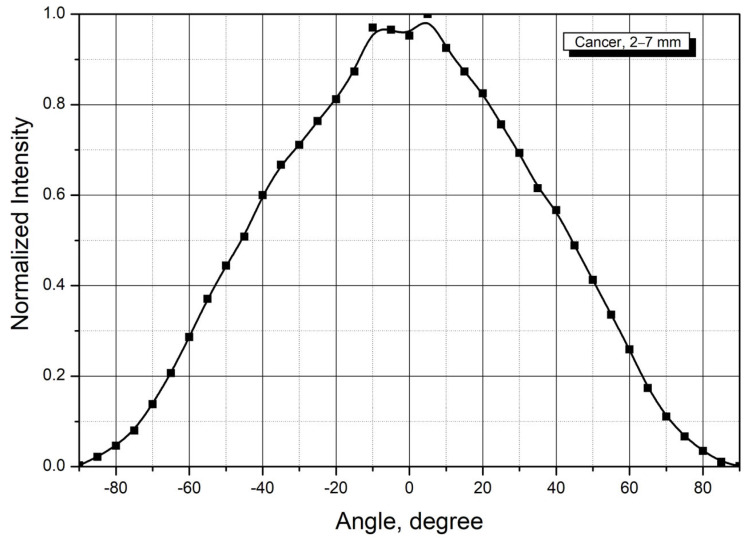
Angular scattering distributions from tumor tissue samples. Note: average values for 13 samples with thicknesses from 1.8 to 7.3 mm.

Angular scattering distributions for tumor tissue samples, measured using the described methodology, are presented in Figure 5.

**Figure 6:**
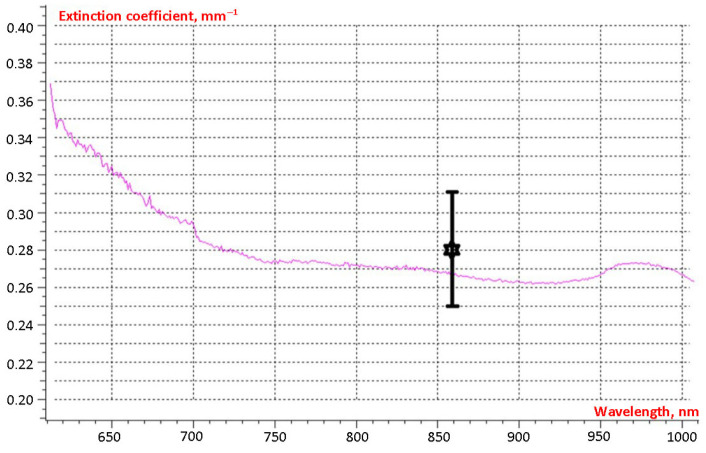
Results of spectral attenuation coefficient measurements for breast tumor tissue sample № 2024-11-19. The result obtained from the scattering measurement apparatus, including its error range, is marked with an asterisk.

The scattering distribution from tumor tissue is similar to that observed in healthy tissue. Analysis of the results demonstrates that Equation (2) can be used to estimate the radiation dose for tumor depths exceeding a few millimeters when propagation occurs along the laser beam axis. For sample № 2024-11-19, the tumor tissue attenuation coefficient was determined using a spectrophotometer. The results are presented in Figure 6. As with the healthy tissue measurements, the results from both methods agree within the experimental uncertainty.

All tumor tissue samples exhibited higher attenuation coefficients than normal tissue. Attenuation coefficients ranged from 0.28 to 0.48 mm^−1^, with a mean value 0.39 mm^−1^.

### 4.4. Skin Tissue Testing

**Figure 7:**
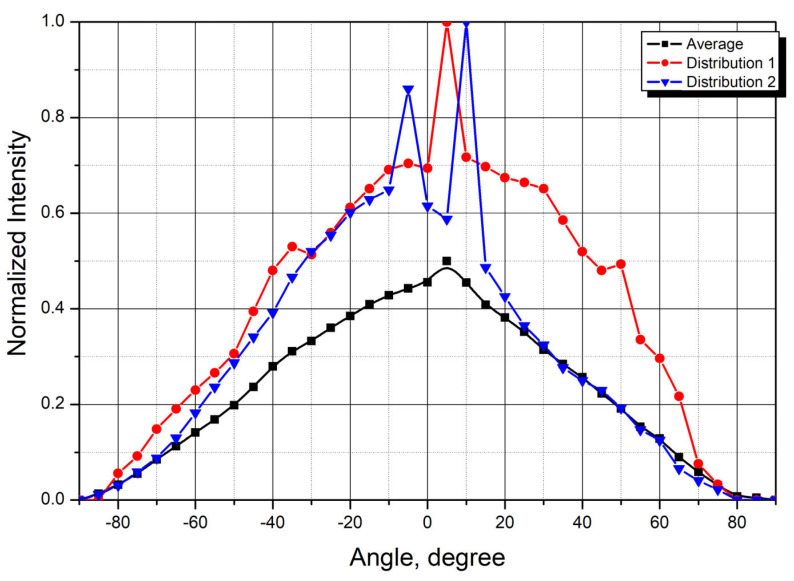
Angular scattering distributions from skin tissue samples. Average values and representative individual measurements.

Figure 7 presents the averaged scattering distribution from 12 skin samples, along with two representative individual measurements from samples of 5.9 mm thick sample (Distribution 1) (№ 2025-01-23) and the 4.6 mm thick sample (Distribution 2) (№ 2024-09-17).

The mean attenuation coefficient was 0.45 mm^−1^. These results agree with published values for Fitzpatrick skin phototypes I, II, and III [34].

### 4.5. Adipose Tissue Testing

**Figure 8:**
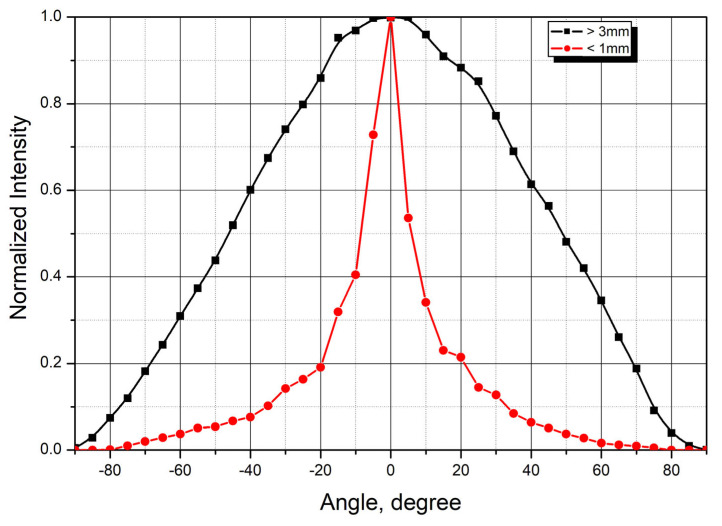
Angular scattering distributions from adipose tissue samples of varying thickness. Average values.

Figure 8 presents angular scattering distributions from adipose tissue samples of varying thicknesses. Similarly to breast tissue, thin adipose samples exhibit high directivity in the scattered radiation. For samples thicker than 3 mm, the scattered radiation shows no directivity, indicating predominantly diffuse scattering.

The mean attenuation coefficient for subcutaneous adipose tissue was 0.44 mm^−1^. These values are consistent with published data providing approximation equations for scattering coefficients across a wide spectral range based on measurements [35,36]. The obtained results of measuring the attenuation coefficient of adipose tissue parameters were close to the data of other studies as well as to the experimental results for subcutaneous fat of porcine tissue (~0.39 mm^−1^) [37].

### 4.6. Laser Attenuation Coefficients and Dose Calculation Algorithm

Measurements were performed for samples with a thickness of at least 2–3 mm, when the influence of thickness on the measurement results is negligible.

To minimize thickness-related effects on the measurements, only samples with a thickness of at least 2 mm were used. The attenuation coefficients for all studied breast tissue samples are summarized in Table 1.

The results enable estimation of both the radiation dose delivered to the tumor and the dose absorbed by tissues along the laser propagation axis.

Based on the experimental data, a program was developed to calculate the absorbed dose for pathological lesions at any location in the body [20,33].

This program determines attenuation coefficients for all breast tissue types encountered along the laser beam path.

**Figure 9:**
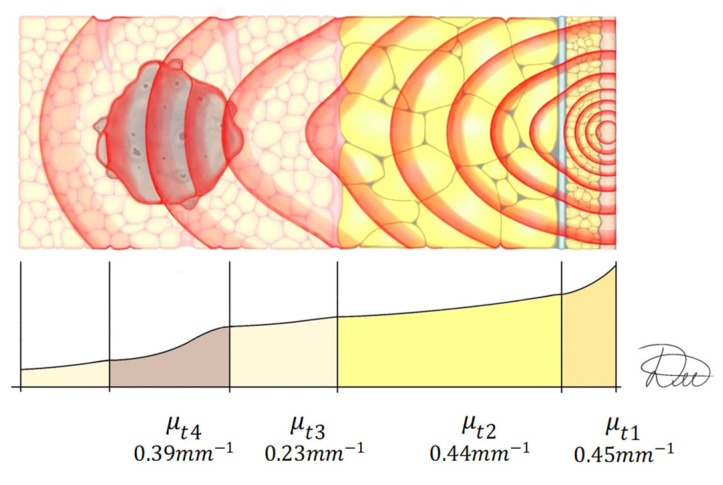
Layer-wise propagation of laser radiation in breast tissue. Attenuation coefficients: skin ($\mu_{t1}$), subcutaneous adipose tissue ($\mu_{t2}$), breast tissue ($\mu_{t3}$), tumor tissue ($\mu_{t4}$). Schematic representation of laser irradiation of the breast. Original illustration by the authors.

The calculated output includes radiation energy densities at tissue boundaries and absorbed energy densities in each tissue layer. The required input parameters are tissue thickness, absorption coefficients, laser operating mode, laser power, and irradiation duration. Mean attenuation coefficients were calculated from these data (Figure 9).

**Figure 10:**
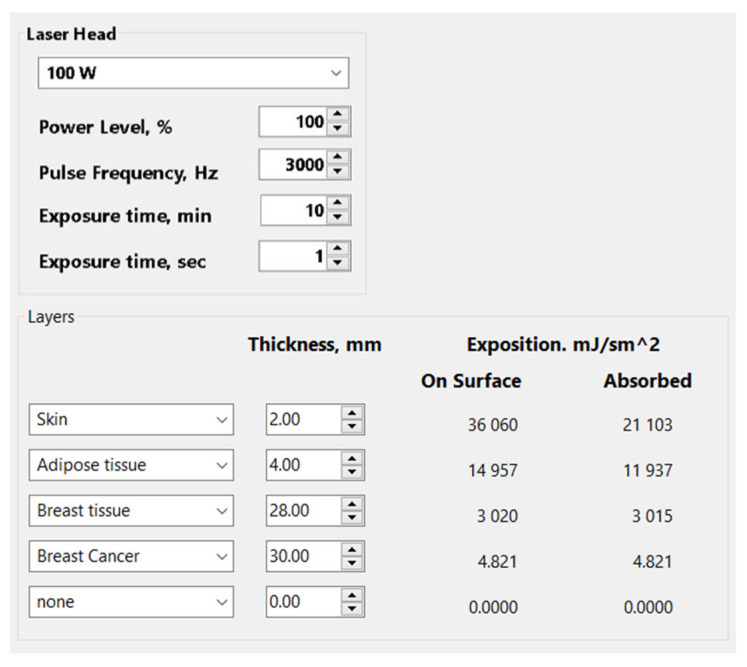
Software interface for calculating absorbed laser dose in biological tissues.

A program interface was developed to calculate the absorbed dose. The program uses laser radiation parameters (power, pulse frequency, irradiation time) and tissue attenuation coefficient values as input (Figure 10).

The software program includes the capability to input and modify tissue parameters for all layers along the laser beam path, including skin, adipose tissue, breast glandular tissue, and tumor tissue.

The energy density of laser radiation (exposure) falling on the tumor interface at time *t* when irradiated with a sequence of laser pulses of duration *τ*, and frequency *F* is related to the intensity as follows:

(3) $$Q_{N}=I_{0}Ft\tau \prod_{i=1}^{N}\left(1-R_{i}\right)e^{-\mu_{ti}x_{i}}$$

where *R_i_*, $\mu_{ti},x_{i}$—reflection coefficient, attenuation coefficient and thickness of *i*-th layer, *I*_0_—output radiation intensity of the therapeutic laser, *N*—total number of tissue layers prior to the tumor to be irradiated.

Future versions of the program will be extended to calculate the three-dimensional distribution of laser intensity within tissue. This will enable dose assessment not only along the beam axis but also in surrounding tissues.

**Figure 11:**
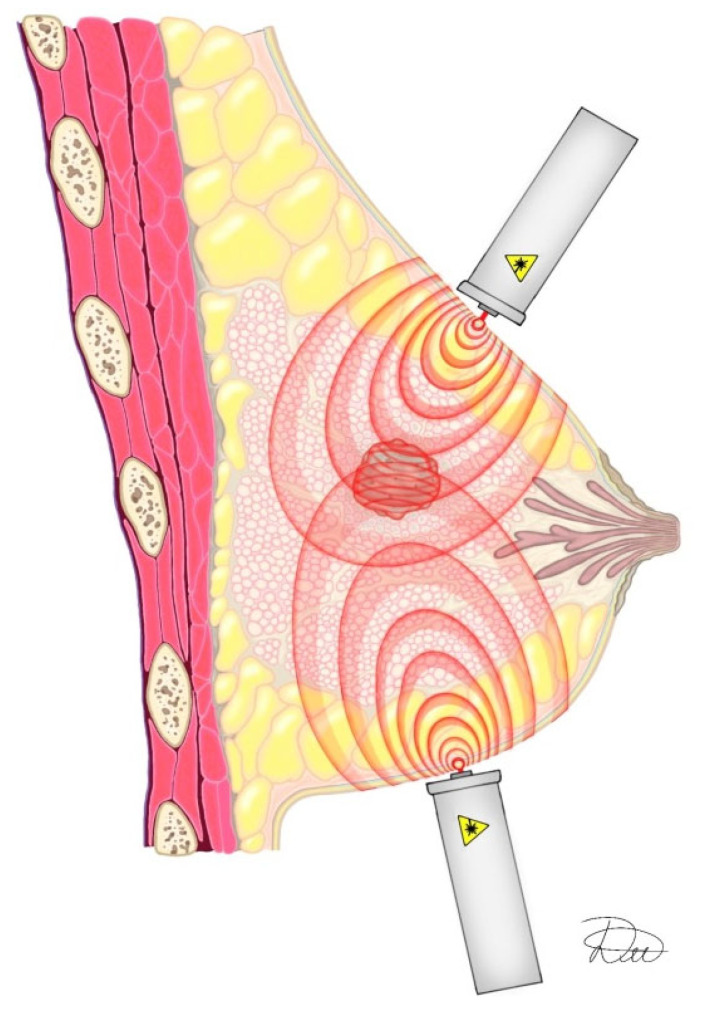
Pulse propagation during simultaneous irradiation from two treatment sites. Schematic representation of laser irradiation of the breast. Original illustration by the authors.

Since 1992, therapeutic laser techniques have allowed treatment from single or multiple anatomical sites, depending on lesion location. This flexibility enables optimized dose delivery to the tumor while minimizing exposure to healthy tissue. For deeply located breast lesions, treatment typically involves irradiation from two separate areas (Figure 11).

Absorbed dose is calculated individually for each irradiation site. Dose calculations primarily use ultrasound, mammography, and CT imaging data. MRI is used in younger patients, for small lesions, and in patients with metallic implants.

## 5. Discussion

**Figure 12:**
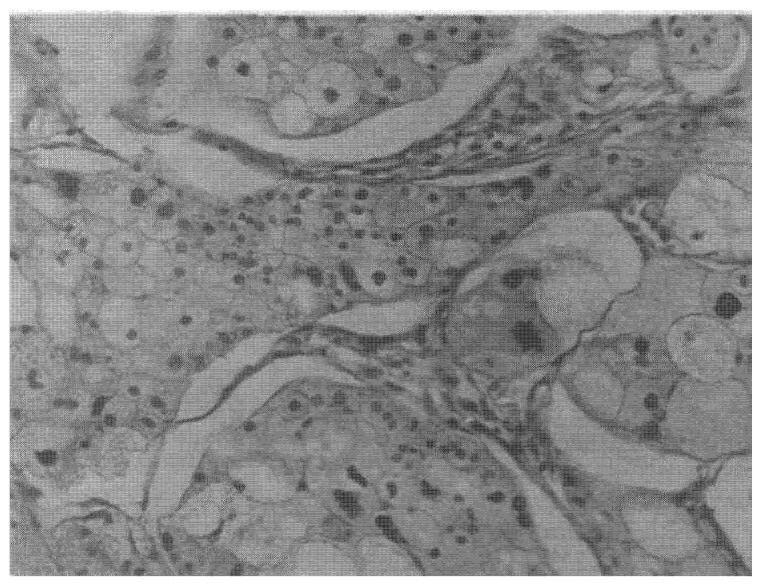
Histopathology of invasive breast carcinoma showing treatment-induced tumor necrosis. Note: marked dystrophic changes, cytoplasmic enlargement with vacuolization, and pathologic mitotic figures (magnification 400, hematoxylin and eosin).

Laser therapy has been used clinically since 1991. Today, it serves both as a primary treatment modality and as an adjunct to conventional therapies. As a standalone treatment, it is primarily employed in advanced disease when conventional methods have failed. It is also increasingly combined with surgery, chemotherapy, and radiotherapy. In Stage IV patients, survival increased up to 3–4 fold, with marked improvements in quality of life. Laser therapy proved most effective for breast and brain cancers. Preoperative laser therapy induced therapeutic pathomorphosis in Grade III tumors (Figure 12). Histological examination revealed extensive tumor necrosis, with surviving malignant cells displaying significant degenerative changes. Cytoplasmic vacuolization was prominent, indicating treatment-induced cellular damage (Figure 12).

In patients with stage IIa–IIIa disease receiving perioperative laser therapy (preoperatively and up to 24 months postoperatively), 10-year survival improved significantly: 10.71% for IIa st. and 5.6% for IIIa st., compared to surgery alone. Although these data were obtained over 20 years ago (1988–2001), they established the foundation for subsequent research into laser-based cancer therapy.

A major limitation of early dosimetry calculations was their inability to account for optical tissue properties. Previous investigations failed to consider tissue thickness effects, beam scattering characteristics, or angular-dependent scattering at different propagation angles.

To accurately characterize tissue scattering coefficients, two complementary measurement approaches were employed: quantification of laser absorption at varying tissue thicknesses and assessment of angular-dependent scattering distributions.

Based on these findings, a methodology was developed to calculate absorbed laser dose using measured tissue scattering coefficients combined with imaging data (mammography, ultrasound, CT, and MRI). A corresponding software program was created to individualize dose calculations for tumors at different anatomical locations. This approach enables selection of optimal radiation doses tailored to each patient and lesion, maximizing therapeutic efficacy while minimizing exposure to healthy tissue.

## 6. Conclusions

1. Laser radiation attenuation coefficients were comprehensively characterized for various breast tissue types across multiple thicknesses and beam propagation angles. Tumor tissue exhibited scattering coefficients approximately 16.5% higher than normal breast tissue.
2. A quantitative methodology was developed to accurately calculate laser-absorbed dose based on measured tissue scattering coefficients and diagnostic imaging data (ultrasound, mammography, CT, and MRI).
3. This methodology was implemented as a clinically applicable software program, enabling personalized dose calculations for breast tumors of any size and anatomical location. This approach facilitates optimization of laser therapy parameters for individual patients, maximizing therapeutic efficacy while minimizing exposure to healthy tissue.

## Figures and Tables

**Table 1:** Attenuation coefficients measurement results.

Sample	№	Attenuation Coefficient, mm^−1^	Notes
Breast tissue	2024-05-11	−0.26 ± 0.03	
Breast tissue	2024-05-14	−0.17 ± 0.03	
Breast tissue	2024-09-10-1 2024-09-10-2	−0.49 ± 0.05 −0.30 ± 0.03	
Breast tissue	2024-09-12	−0.26 ± 0.03	
Breast tissue	2024-09-13	−0.23 ± 0.04	
Breast tissue	2024-09-17	−0.27 ± 0.08	
Breast tissue	2024-10-07	−0.19 ± 0.02	
Breast tissue	2024-10-08	−0.27 ± 0.02	
Breast tissue	2024-11-19	−0.23 ± 0.02	
Breast tissue	2025-01-14-1	−0.22 ± 0.02	
Breast tissue	2025-01-20	−0.21 ± 0.02	
Breast tissue	2025-01-23	−0.20 ± 0.03	
Adipose	2024-05-27-1 2024-05-27-2	−0.48 ± 0.09 −0.33 ± 0.08	Invasive ductal carcinoma, Invasive ductal carcinoma
Breast tumor	2024-09-16	−0.34 ± 0.09	
Breast tumor	2024-09-17	−0.38 ± 0.06	Invasive ductal carcinoma
Breast tumor	2024-10-10	−0.43 ± 0.04	Invasive ductal carcinoma
Breast tumor	2024-10-15	−0.14 ± 0.01	Invasive ductal carcinoma
Breast tumor	2024-11-19	−0.28 ± 0.03	Invasive ductal carcinoma Comparison with spectrophotometer data
Retroperitoneal tissue	2024-05-15	−0.38 ± 0.09	
Gastrocolic omentum	2024-05-22	−0.29 ± 0.06	
Adipose tissue	2024-10-08	−0.51 ± 0.1	
Skin + breast tissue	2024-09-17	−0.44 ± 0.13	
Epidermis Derma	2024-11-22	−0.58 ± 0.14 −0.20 ± 0.02	
Skin	2025-01-17	−0.48 ± 0.09	
Skin	2025-01-23	−0.29 ± 0.03	
Blood	2024-12-17	−0.97 ± 0.06	

## Data Availability

The original contributions presented in this study are included in the article. Further inquiries can be directed to the corresponding author.

## Abbreviations

LLLT	Low-level laser therapy
CT	Computed tomography
MRI	Magnetic resonance imaging
st.	Stage
Fig.	Figure
cm	centimeter
mm	millimeter
nm	nanometer
